# Assessment of potentially toxic elements in mine tailings and their categorization and prioritization as environmental liabilities in gold mining areas of Antioquia, Colombia

**DOI:** 10.1007/s10653-026-03322-5

**Published:** 2026-07-10

**Authors:** Sebastian Collazo, Esteban Vélez, Juan D. Correa, Juan F. Marín, Juan F. Saldarriaga, Julián E. López

**Affiliations:** 1 Faculty of Architecture and Engineering, Study Group SICA-GeoHealth, Semillero de Investigación SICA, Institución Universitaria Colegio Mayor de Antioquia, Carrera 78 # 65-46, 050034 Medellín, Colombia; 2 Department of Civil and Environmental Engineering, Universidad de los Andes, Carrera 1Este #19A-40, 111711 Bogotá, Colombia

**Keywords:** El Bagre, Environmental management, Heavy metals, Mining waste, Nechí, Zaragoza

## Abstract

In El Bagre, Nechí, Zaragoza, and three other municipalities that comprise the Bajo Cauca region of Antioquia, an estimated total of 28.000 ha have been affected by illegal gold mining, where environmental liabilities such as mine tailings are distributed throughout the area. Urban areas, especially the main urban centers, may be exposed to environmental contaminants and their associated adverse effects. Studies that contribute to the characterization and prioritization of environmental liabilities are necessary to improve environmental management and public policies. The aim of this study was to assess the presence of As, Cd, Cr, and Pb associated with mine tailings in El Bagre, Nechí, and Zaragoza and to implement a methodology for their characterization and prioritization through the calculation of risk index (RI) values. The RI considered both the concentrations of potentially toxic elements (PTEs) and the proximity of mine tailings to sensitive receptors, including urban areas and environmental features such as surface water bodies. Spatial distribution maps of PTE concentrations and RI values were subsequently developed to identify priority environmental liabilities for management. One of the main results of the study is the identification of five mining tailings as prioritized environmental liabilities for environmental management, which was unknown prior to the completion of this study. No environmental liabilities classified as high risk were identified in any of the three municipalities. In El Bagre, 9/11 environmental liabilities were classified as low risk, while 2/11 were classified as medium risk. In Zaragoza, 4/7 environmental liabilities were classified as low risk and 3/7 as medium risk. In Nechí, 6/6 environmental liabilities were classified as low risk. To the best of our knowledge, this study is the first to apply a methodology for the classification and prioritization of mining tailings in the study area. Another important result was the spatial mapping of the distribution of PTEs across the three municipalities, as this outcome allows the spatial identification of PTE concentrations and serves as an input for environmental planning of the territories. In conclusion, the results provide scientific evidence that will contribute to the development of a methodology for the categorization and prioritization of environmental liabilities associated with gold mining, serving as a resource for environmental authorities and guiding decision-making based on scientific and technical evidence.

**Supplementary Information:**

The online version contains supplementary material available at 10.1007/s10653-026-03322-5.

## Introduction

Formalized gold mining has historically played a key role in the development of many countries around the world, for instance, in African countries such as Ghana, Mali, Zimbabwe, and Tanzania; in Asian countries such as Philippines, Indonesia, Kazakhstan, and Uzbekistan; and in Latin American countries such as Peru, Brazil, Mexico, Venezuela, Chile, and Colombia (Kumah, 2006) According to data from the World Gold Council, global gold production in 2025 was estimated at approximately 3.670 metric tons. Formalized gold mining has increasingly adopted sustainable practices that not only generate economic value but also promote environmental stewardship and social well-being (Kumah, 2006). In Colombia, formalized gold mining is an economic activity of significant importance, which has driven economic development (Baena & Mendoza, 2021). Economic data from 2025 indicate that gold exports accounted for approximately 39% of the mining sector’s exports, equivalent to about USD 3.5 billion FOB (Agencia Nacional de Minería & UPME, 2025). Overall, the mining sector represented approximately 1% of the country’s GDP in 2025 (Agencia Nacional de Minería & UPME, 2025). In the country, gold mining is distributed across approximately 70% of the national territory and serves as the primary livelihood for nearly 15.000 families, representing approximately 165.000 people (Agencia Nacional de Minería & UPME, 2025; Rodríguez-Zapata & Agudelo, 2024).

However, not all gold extraction processes are formalized and some activities are carried out through illegal practices, which have been associated with environmental degradation and governance challenges (Vélez-Torres & Vanegas, 2022). For instance, there are reports indicating that 20% of global gold production is associated with informal or illegal mining (Fermet-Quinet et al., 2025). In Africa, it is estimated that approximately 30% of the continent’s gold mining activities are carried out illegally, for example, in countries such as Ghana, it is estimated that 43% of gold mining activities operate illegally (Manduna, 2025; Stærfeldt & Stacey, 2025). In Latin America, data from Peru indicate that approximately 28% of gold mining activities are illegal (Elera-Gonzales et al., 2025). Data from Brazil indicate that approximately 71% of gold mining activities, especially those concentrated in the Amazon region, are illegal mining operations (Cortinhas Ferreira Neto et al., 2024). In Colombia, it is estimated that only 13% of gold mining operations are legally authorized (CEPAL, 2000; Vélez-Torres & Vanegas, 2022), while the remaining 87% operate outside the regulatory framework and the oversight of control authorities. This situation has led to the occurrence of environmental liabilities in several territories highly impacted by illegal mining. In Colombia, only about 70 environmental liabilities linked to illegal mining have been officially recorded; however, the vast majority of liabilities associated with this activity remain undocumented and unstudied (Rodríguez-Zapata & Ruiz-Agudelo, 2021). It is estimated that 47% of the environmental liabilities associated with gold mining fall under this category (Rodríguez-Zapata & Ruiz-Agudelo, 2021).

The scarcity of studies on, this issue constrains the capability of environmental authorities and other stakeholders to properly manage the environmental concerns. Further aggravating the problem, some of these liabilities are classified as orphan environmental liabilities, which could be defined as environmental damage for which no responsible party can be identified, either because liability cannot be established or, if it is established, the responsible parties are unable to assume the costs of remediation. Gold mining environmental liabilities include facilities, soils affected by spills, disturbed watercourses, machine shops, tool storage areas, ore storage sites, and waste deposits. In the latter case, the waste refers, for instance, to mining tailings (Salgado-Almeida et al., 2022, 2024; López et al., 2025).

Mining tailings are among the most recurrent forms of orphan environmental liabilities resulting from illegal gold mining, due to the lack of environmental controls and the abandonment of waste. Mining tailings are a by-product of the mineral extraction process, consisting of finely ground rock, water, and processing reagents left over after valuable minerals are separated from mined ore (Espin, 2023; Salazar et al., 2024). The inadequate management of mining tailings poses significant environmental and social risks, such as water and soil contamination, as well as serious health hazards, including lung cancer, skin cancer, kidney damage, skin irritation, brain damage, diabetes, cardiovascular disease, reproductive dysfunction, and anemia (Banerjee et al., 2023; Wang et al., 2025). The potential health risk and environmental damage associated with mining tailings are mainly related to the presence of potentially toxic elements (PTEs) such as As, Cd, Pb, Cr, and Hg, among others (Salazar et al., 2024; Timsina et al., 2022). However, in regions such as Antioquia, which accounts for 40% of Colombia’s gold mining activity, studies of this kind have been scarce, particularly in the sub-region of Bajo Cauca Antioquia, a territory historically affected by mining. This sub-region is characterized by the presence of both formal and illegal mining activities (Baena & Mendoza, 2021). It is estimated that approximately 28.000 ha in this area have been disturbed as a result of gold mining (Berrio-Giraldo et al., 2026). In the area, there is evidence of tailings deposits resulting from illegal mining activities. These deposits are considered orphaned environmental liabilities.

According to the literature reviewed, there is a lack of studies in Colombia that systematically assess alternatives for the classification and prioritization of environmental liabilities, particularly methodologies that incorporate the risks associated with urban environments located in territories affected by illegal gold mining activities In contrast, in other American countries with comparable challenges from illegal gold mining, such as Ecuador, research has been carried out to characterize and prioritize environmental liabilities in mining-impacted areas (Salgado-Almeida et al., 2022). In these researches, the environmental risk related to environmental liabilities existing in three artisanal and small-scale gold-mining areas of Ecuador was evaluated. The results helped identify certain areas as high-risk zones, which should be prioritized for remediation. On the other hand, studies in Brazil have begun to systematically consolidate the social, environmental, and economic needs required for the management of mining environmental liabilities (Da Silveira Barros & França, 2025). Another example is the application of decision-grade risk and cost mapping methodologies for illegal gold mining in Costa Rica, to support prioritization, phased remediation portfolios, and uncertainty-aware policy ranking (Navarro Jiménez, 2026). In other study conducted in French Guiana, a multi-criteria classification of mining projects for risk assessment at the territorial scale was developed (Scammacca et al., 2021). These studies provide a reference framework that supports the relevance of developing and applying decision-making methodologies as essential tools to promote effective environmental management of environmental liabilities by the competent authorities.

Therefore, this study aims to assess the presence of PTEs associated with mine tailings in three areas affected by illegal gold mining in Colombia and to implement a methodology for their characterization and prioritization. Tailings samples were collected and analyzed to determine the concentrations of As, Cd, Pb, and Cr. Geochemical indices were then calculated to evaluate contamination levels, and spatial distribution maps were generated to visualize PTEs concentrations. To characterize and prioritize the risk associated with mine tailings, the risk index was applied, followed by the construction of maps showing the spatial distribution of risk index values. The results of this study provide novel insights into contamination in areas impacted by gold mining and represent a useful tool for the environmental management of mining-related liabilities.

## Materials and methods

### Study area

**Fig. 1:**
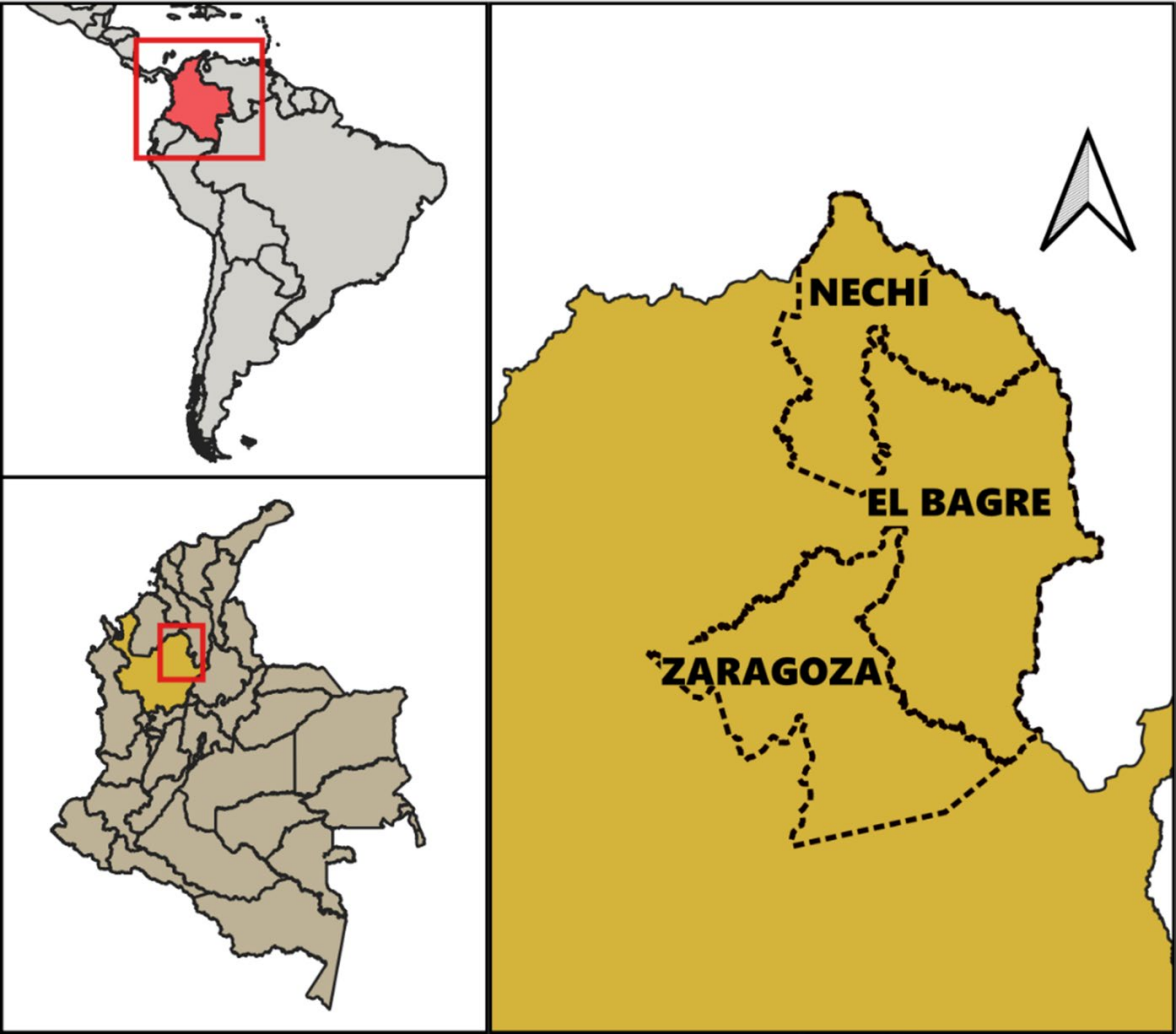
Municipalities of Nechí, El Bagre, and Zaragoza, Antioquia, Colombia

**Fig. 2:**
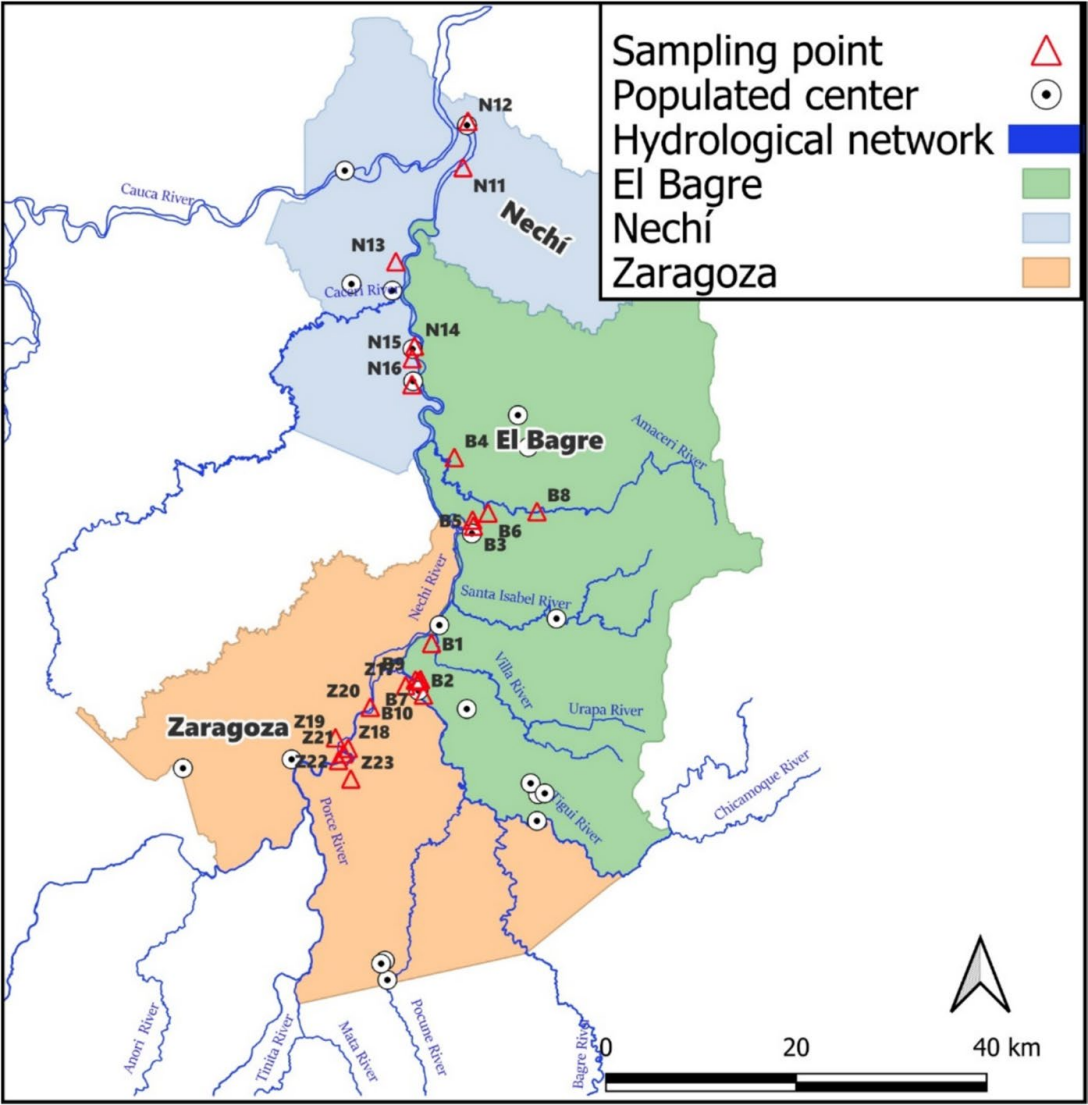
Study area, sampling points, population centers, and hydrological network

The municipalities of Zaragoza, El Bagre, and Nechí are located in the Bajo Cauca sub-region, Antioquia, Colombia (Fig. 1) and have an area of 914, 1.067, and 1.563 km^2^, respectively. The main rivers crossing the region are the Cauca, Nechí, Bagre, and Tigüí rivers (Agudelo-Echavarría et al., 2020) (Fig. 2). The sub-region is mainly composed of Quaternary alluvial deposits consisting of sands, silts, clays, and auriferous gravels associated with the fluvial dynamics (Fig. S1). These deposits have historically favored alluvial gold exploitation (Fig. S1). Additionally, high-grade metamorphic units, as well as tonalitic and granodioritic intrusions related to vein-type gold mineralization, outcrop within the Bagre–Nechí mining district (Fig. S1). Relevant tectonic structures have also been identified in the area (Londoño-Herrera et al., 2009) (Fig. S1). The main soil orders correspond to Entisols and Inceptisols developed over recent sediments, with textures ranging from fine to coarse and generally low to moderate fertility (IGAC, 2015) (Figs. S1 and S2). In older and more weathered sectors, acidic Ultisols with low natural fertility are also present (IGAC, 2015) (Fig. S2). In several areas of the Bajo Cauca region, problems related to erosion, compaction, and loss of productive capacity have been reported due to intensive gold mining activities and the alteration of soil profiles (IGAC, 2015). The area is characterized by a humid tropical climate with annual precipitation ranging approximately between 2.400 and 4.000 mm. Rainfall is concentrated mainly between April and November, while short dry periods occur between July and August. High precipitation levels and the influence of the Cauca, Nechí, Tigüí, and Bagre rivers promote frequent flooding processes and flash floods, particularly in alluvial plains and mining exploitation areas.

Demographic data for the municipalities of Nechí, El Bagre, and Zaragoza indicate an approximate population of 25,000, 55,000, and 28,000 inhabitants, respectively (DANE, 2018). In the municipality of Nechí, 45% of the population is located in urban areas, while the remaining 55% is distributed in rural areas. In the municipality of El Bagre, the largest proportion of the population, approximately 65%, is concentrated in urban areas, whereas 35% is located in rural areas. In the municipality of Zaragoza, 52% of the population is located in urban areas and 48% in rural areas. In Nechí, approximately 51% of the population is male and 49% female, while in El Bagre the distribution is approximately 52% male and 48% female. In Zaragoza, the gender distribution is nearly balanced, with approximately 50% male and 50% female population (Alcaldía Municipal de El Bagre, 2023; Alcaldía Municipal de Nechí, 2023; Alcaldía Municipal de Zaragoza, 2023). The population structure across the three municipalities is predominantly young, with a high proportion of inhabitants under 35 years old and a significant working-age population associated mainly with artisanal and small-scale gold mining, agriculture, livestock farming, and commercial activities. In these municipalities, the main economic activities are artisanal and small-scale gold mining, livestock farming, agriculture, commerce, and local trade (Alcaldía Municipal de El Bagre, 2023; Alcaldía Municipal de Nechí, 2023; Alcaldía Municipal de Zaragoza, 2023).

### Mine tailings sampling

The samples were collected from mine tailing deposits associated with illegal mining processes, which are spread throughout each municipality (Fig. 2). In the municipality of Nechí, a total of 6 composite samples were obtained. In El Bagre, 10 composite samples were collected, and in Zaragoza, a total of 7 composite samples were collected. All sampled tailings deposits had an area smaller than 2 ha. The number of composite samples collected in each municipality depended on the number of tailings deposits that were accessible for sampling. Accessibility to the tailings deposits was a critical aspect of the study, as some areas did not have safe access routes, while others had access restrictions due to the presence of illegal armed groups. For each composite sample, 30 subsamples (30–40 g) were collected and combined to form a single composite sample. The sampling methodology helps minimize sampling error and ensures representativeness (Smith et al., 2006). The subsamples were collected at a depth of 30 to 40 cm (Salgado-Almeida et al., 2024). Each composite sample weighed 1000 g. Subsequently, the composite samples were air-dried and then sieved (< 2 mm).

### PTEs concentrations

For the determination of concentration of PTEs, 1.0 g of sample was subjected to acid digestion (HNO_3_:HCl) in an Ethos One Milestone helping Chemist microwave. In brief, 10 ml of HNO_3_ and 30 ml of HCl were added. The temperature of the samples was raised to 175 °C in approximately 5.5 min and remained at 175 °C for an additional 4.5 min, following USEPA method 3051A. After digestion, the samples were diluted with ultrapure water and passed through a 0.45 μm membrane filter, subsequently the filtrates were used for the determination of PTEs by inductively coupled optical emission spectrometry using an Agilent Technologies 5100 ICP-OES equipment (ICP-OES).

### Quality assurance and quality control protocols

Quality assurance and quality control protocols were incorporated into the procedure for measuring As, Cd, Pb, and Cr concentrations. Four certified synthetic standard solutions for trace elements (Millipore Sigma) were used for ICP-OES calibration. The calibration curve was accepted with a correlation coefficient (R^2^) greater than 0.9996, following the specifications of the original source. The LOD (mg L^−1^) for Pb, As, Cr, and Cd were 0.089, 0.028, 0.041, and 0.007, respectively. The LOQ (mg L^−1^) for Pb, As, Cr, and Cd were 0.015, 0.09, 0.013, and 0.003, respectively. Certified reference material (CRM) BCR® 701 Lake Sediment (European Commission´s Joint Research Centre – JCR), was included in each digestion/extraction batch for quality assurance. Element recoveries from the samples, calculated relative to certified concentrations, ranged from 96 to 103% for Pb, 94 to 102% for As, 93to 105% for Cr, and 90 to 101% for Cd. Duplicate samples were analyzed to assess reproducibility. Certified synthetic standards (Millipore Sigma) and a blank (deionized water) (Milli-Q water, 18.2 MΩ) were also included in each batch for quality control. All reagents used were of analytical grade, and all glassware was soaked overnight in a 3% (v/v) HNO_3_ acid bath prior to use.

### Geoaccumulation index

The geoaccumulation index (Igeo) for the PTEs were determined using Eq. 1. Although the Igeo was originally introduced by (Müller et al., 1979) for assessing contamination levels in river sediments, its application has been widely extended to terrestrial environments, including soils, sediments and tailings (Loska et al., 2004) (Kowalska et al., 2018; Uugwanga & Kgabi, 2020a; Fernández-Martínez et al., 2024).

1 $${\text{Igeo }} = Log2 \left( {\frac{{{\mathrm{XC}}}}{{1.5*{\mathrm{CB}}}}} \right)$$

where XC is the concentration of PTE, CB is the value of geochemical background, and 1.5 is the background matrix correction factor used to compensate for natural fluctuations (Fernández-Martínez et al., 2024). In this study, for each PTE the CB value of Colombia was used: As (12.2 mg kg^−1^), Cd (2.5 mg kg^−1^), Pb (22.8 mg kg^−1^), and Cr (121 mg kg^−1^) (López et al., 2025). Igeo index ranges from: Igeo < 0 refers to uncontaminated, 0 < Igeo < 1 refers to low contamination, 1 < Igeo < 2 refers to moderately contaminated, 2 < Igeo < 3 refers to highly contaminated, 3 < Igeo < 4 refers to heavily contaminated, 4 < Igeo < 5 refers to very heavily contaminated, and Igeo > 5 refers to extremely contaminated (Li et al., 2014).

### Potential ecological risk index

The potential ecological risk index (PERI) was determined as the sum of single hazard factors (Eq. 2). This method was initially introduced by (Hakanson, 1980). This index has been previously used to determine ecotoxicological risk due to the presence of PTEs in different environmental matrices, including mining waste (Azizi et al., 2025; Kowalska et al., 2018; Salazar et al., 2024). Since this index uses PTE concentrations and relates them to a reference toxicological response factor, it can be applied regardless of the environmental matrix to assess the degree of ecological risk from PTE concentrations. Consequently, it is applicable to water, air, and soil (Kowalska et al., 2018; Ngole-Jeme & Fantke, 2017).

2 $$PERI = \mathop \sum \limits_{{\phantom{a}}}^{{\phantom{a}}} {\mathrm{Eir}} = \mathop \sum \limits_{{\phantom{a}}}^{{}} {\text{Tir* }}\frac{{{\mathrm{XC}}}}{{{\mathrm{CB}}}}$$

where Eir is the single hazard factor, Tir is the toxic response factor por each PTEs, Table 1 summarizes the values of Tir used (Hakanson, 1980a) (Salazar et al., 2024). XC is the concentration of PTE, CB is the value of geochemical background. In this study, for each PTE the CB value of Colombia was used: As (12.2 mg kg^−1^), Cd (2.5 mg kg^−1^), Pb (22.8 mg kg^−1^), and Cr (121 mg kg^−1^) (López et al., 2025). According to the values of the Eir in: Low risk < 40, Moderate risk 40 ≤ Eir < 80, Considerable risk 80 ≤ Eir < 160, High risk 160 ≤ Eir < 320, and very high risk Eir ≥ 320. The values of the PERI risk index are interpreted as: low risk, PERI < 50, Moderate risk 50 ≤ PERI < 200, Considerable risk 200 ≤ PERI < 300, and high-risk PERI ≥ 300 (Hakanson, 1980b).

**Table 1:** Toxic response factor (Tir) for contamination by each potential toxic element

Tir*	Value
As	10.0
Cd	30.0
Pb	5.0
Cr	2.0

### Categorization and prioritization of environmental liabilities

Mine tailings were categorized and prioritized using the risk assessment methodology proposed by the Spanish Geological Survey (Salgado-Almeida et al., 2024). Within this framework, the associated risk was quantified using the Risk Index (RI), calculated as the product of the Probability Index (PI) and the Severity Index (IS). The PI was determined using Eq. 3, which incorporates factors such as proximity to water bodies (PR), toxicity of mining wastes (FTOX), and the presence of unprotected surfaces (FSD). The evaluation criteria for PR are presented in Table 2. The FTOX value was derived from the Average Hazard Quotient (AHQ), as defined in Eq. 4, where XC represents the concentration of PTEs. MPLi denotes the maximum permissible level for PTEs, and n is the number of PTEs in the sample with concentrations exceeding their respective regulatory limits. The maximum permissible values reported by Salgado-Almeida et al. (2022) for Ecuador were used, as Colombia does not have established regulatory standards for threshold values of PTEs in soils and/or mining tailings. Table 2 summarizes the criteria used to assign PR, FTOX, and FSD values.

**Table 2:** Criteria of parameters for the probability index (IP) and severity index (IS) determination

Parameter	Criteria	Value
Proximity factor to water bodies (PR)	D ≤ 50 m 50 < D < 500 m D ≥ 500 m	PR = 1.0 PR = -0.0022 * D + 1.1 PR = 0.0
Toxicity FACTOR (FTOX)	AHQ ≤ 400 AHQ > 400	FTOX = 0.0125 * AHQ FTOX = 5.0
Unprotected surface factor (FSD)	SEX ≤ 2 ha SEX > 2 ha	FSD = 0.5 * SEX FSD = 1.0
Exposed population to toxic elements factor (PEX)*	SP ≤ 50 SP > 50	PEX = 0.1 * SP PEX = 5.0
Exposure factor (FSUP): FSUP-PO for population and FSUP-NA for environment	DD ≤ 100 m 100 < DD ≤ 5000 m DD > 5000 m	FSUP = 1.0 FSUP = (-0.0002 * DD) + 1 FSUP = 0.0
Vulnerability factor of the exposed population (VP)	Use of very highly vulnerable water Highly vulnerable water Use of vulnerable water Use of low vulnerable water Use of water very little vulnerable	VP = 5.0 VP = 4.0 VP = 3.0 VP = 2.0 VP = 1.0
Ecological vulnerability factor (VE)	Very highly vulnerable resources and ecosystems Highly vulnerable resources and ecosystems Vulnerable resources and ecosystems Low vulnerable resources and ecosystems Very low vulnerable resources and ecosystems	VE = 5.0 VE = 4.0 VE = 3.0 VE = 2.0 VE = 1.0

^*^Considering a maximum radius of 5 km from the tailing deposit. SEX = exposed area of the tailing deposit in ha. D = distance from tailing deposit to water bodies. AHQ = average hazard quotient. SP = supplied people with surface water. DD = distance from the pollutant load in surface water body to the population (for FSUP-PO) or environmental interest areas (for FSUP-NA)

The IS was used to assess the potential impacts of contamination on both the human population (IS-PO) and the natural environment (IS-NA), as defined by Eqs. 5 and 6, respectively. The IS-PO was based on three factors: the proportion of the population exposed to toxic elements (PEX), the population exposure factor (FSUP-PO), and the vulnerability of the exposed population (VP). In turn, the IS-NA was determined using the environmental exposure factor (FSUP-NA) and the ecological vulnerability factor (VE). The criteria applied to evaluate each factor are presented in Table S2.

3 $$IP=PR*FTOX*FSD$$

4 $$AHQ = \frac{1}{n}*\sum\nolimits_{i = 0}^{n} {\frac{{{\mathrm{XC}}}}{{{\mathrm{MPLi}}}}}$$

5 $${\mathrm{IS}} - {\mathrm{PO}} = \, 0.5*{\text{PEX }} + \, 0.5*\left( {{\mathrm{FSUP}} - {\text{PO }}*{\text{ VP}}} \right)$$

6 $${\mathrm{IS}} - {\mathrm{NA}} = {\text{ FSUP}} - {\text{NA }}*{\text{ VE}}$$

Both the PI and the IS are classified into 5 categories: very low (≥ 0 PI or IS < 1), low (≥ 1 PI or IS < 2), moderate (≥ 2 PI or IS < 3), high (≥ 3 PI or IS < 4), and very high (≥ 4 PI or IS ≤ 5). The risk of impact on the population (RI-PO) and the natural environment (RI-NA) was calculated by multiplying the IP by the corresponding IS (IS-PO or IS-NA) (Eqs. 7 and 8). The resulting RI was then categorized into 3 levels: low risk (0 ≤ RI ≤ 5), moderate risk (6 ≤ RI ≤ 15), and high risk (16 ≤ RI ≤ 25).

7 $${\mathrm{RI}} - {\mathrm{PO}} = {\text{ IP }}*{\text{ IS}} - {\mathrm{PO}}$$

8 $${\mathrm{RI}} - {\mathrm{NA}} = {\text{ IP }}*{\text{ IS}} - {\mathrm{NA}}$$

A spatial analysis was performed using QGIS 3.24. A point map was generated to show the spatial distribution of categorized mining tailings. Each point contained spatial coordinates and RI values.

## Results and discussion

### Concentration of PTEs

Table 3 shows the concentrations of PTEs measured in the municipalities of El Bagre, Nechí, and Zaragoza. Substantial variability was observed among municipalities. Zaragoza exhibited the highest mean concentrations of As, Cd, and Pb, whereas El Bagre showed the highest mean Cr concentration.

**Table 3:** Summary statistics of As, Cd, Pb, and Cr concentrations in mining tailings samples from El Bagre, Nechí, and Zaragoza, Antioquia, Colombia

Municipalities	As (mg kg^−1^) Mean ± SD (Min–Max)	Cd (mg kg^−1^) Mean ± SD (Min–Max)	Cr (mg kg^−1^) Mean ± SD (Min–Max)	Pb (mg kg^−1^) Mean ± SD (Min–Max)
El Bagre (n = 10)	94.0 ± 106 (11.8–318)	9.34 ± 6.48 (1.22–24.0)	23.0 ± 13.12 (7.37–41.3)	1844 ± 1022 (201–2998)
Nechí (n = 6)	100 ± 39.70 (77.2–178)	7.90 ± 3.97 (3.58–15.1)	18.16 ± 11.01 (7.86–38.5)	1462 ± 902 (534–2984)
Zaragoza (n = 7)	636 ± 379 (152–865)	11.05 ± 5.55 (2.65–16.9)	22.05 ± 11.24 (9.78–40.1)	1895 ± 541 (1161–2740)

n: number of sampling points. Mean: average concentration. SD: standard deviation. Min: minimum concentration. Max: maximum concentration

Fold-change values were calculated as the ratio between the mean concentrations of PTEs in the compared municipalities (Table 4). The As exhibited the highest variability, with fold-change values exceeding ~ 6 between Zaragoza and the other municipalities, while Cd, Cr, and Pb showed more moderate differences.

**Table 4:** Fold-change comparison of mean PTEs concentrations (mg kg^−1^) among municipalities

Municipalities	As	Cd	Cr	Pb
Zaragoza/El Bagre	6.77	1.18	0.96	1.03
Zaragoza/Nechí	6.31	1.40	1.21	1.30
El Bagre/Nechí	0.94	1.18	1.26	1.26

The concentrations of PTEs detected in the mining tailings may be associated with the geological and mineralogical characteristics of the study area. The geology of the study area is characterized by the presence of Quaternary surficial deposits, clastic sedimentary rocks, volcanic rocks and associated strata, granitoid intrusions, schists and metasedimentary sequences, as well as high-grade metamorphic and migmatitic basement complexes (Fig. S1). In the area, rocks are present that contain mineral phases such as galena (PbS), arsenopyrite (FeAsS), tennantite (Cu_12_As_4_S_13_), proustite (Ag_3_AsS_3_), sphalerite (ZnS) (Cd-enriched variants), chromite (FeCr_2_O_4_), and crocoite (PbCrO_4_), which can naturally contribute to elevated levels of As, Cd, Pb, and Cr in the tailings derived from gold mining in the study area (Alonso et al., 2014; Salazar et al., 2024; López et al., 2025) According to previous studies, the Bajo Cauca subregion of Antioquia is one of the areas in Colombia where elevated concentrations of As have been reported (Alonso et al., 2014). In line with the results, Zaragoza presents the highest As levels compared to El Bagre and Nechí. This can be explained because in Zaragoza, the high-grade metamorphic and migmatitic basement complex and the schists and metasedimentary suite dominate-units that by their nature contain arsenopyrite, proustite, and tennantite as constitutive minerals of the gold-bearing veins. Mining in this area is predominantly hard-rock vein mining, meaning that sulfide-bearing material rich in As is directly extracted and processed, generating tailings with elevated concentrations of this element.

**Fig. 3:**
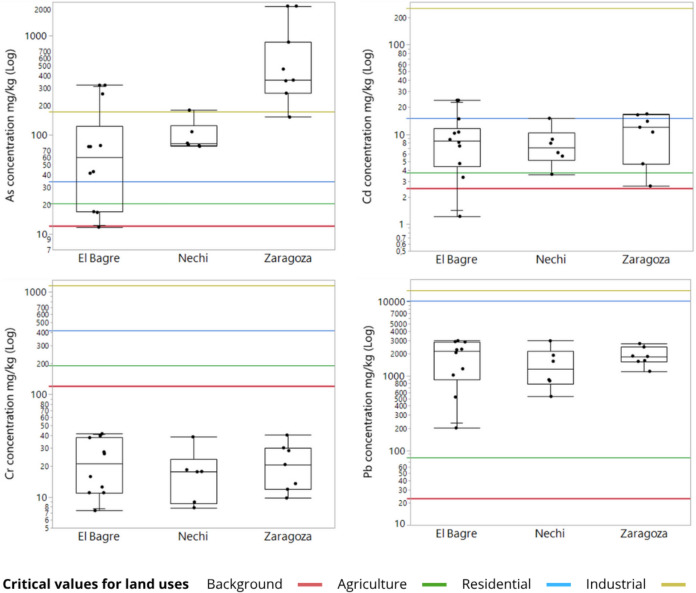
Comparison of PTEs concentrations among the municipalities of El Bagre, Nechi, and Zaragoza, background values, and soil-use guideline thresholds

For a better perception of the PTEs levels detected, these were compared against the critical values established for different land-use categories (Fig. 3). It was observed that As was the most critical PTEs. Approximately 82% of the samples exceeded the residential threshold, and 100% exceeded the industrial threshold. Pb showed a similar behavior, with 100% of the samples exceeding the industrial thresholds and more than 30% surpassing the agricultural and residential values. Cd was present in lower proportions of exceedance. Approximately 17% of the samples exceeded the residential threshold. Cr did not exceed the critical thresholds.

To provide a global perspective, Table S1 presents the concentrations of PTEs reported in mining tailings from different countries, including several countries in Latin America. In general, when comparing PTEs values in mining tailings from different parts of the world, a heterogeneous behavior can be observed. This may be associated with the variability of local geological conditions, as well as site-specific factors such as rainfall, slope, and wind, among others, which influence the mobility of mine tailings and, consequently, the accumulation and concentration of PTEs. This highlights the need for site-specific studies to adequately characterize tailings deposits.

### Geoaccumulation index

**Fig. 4:**
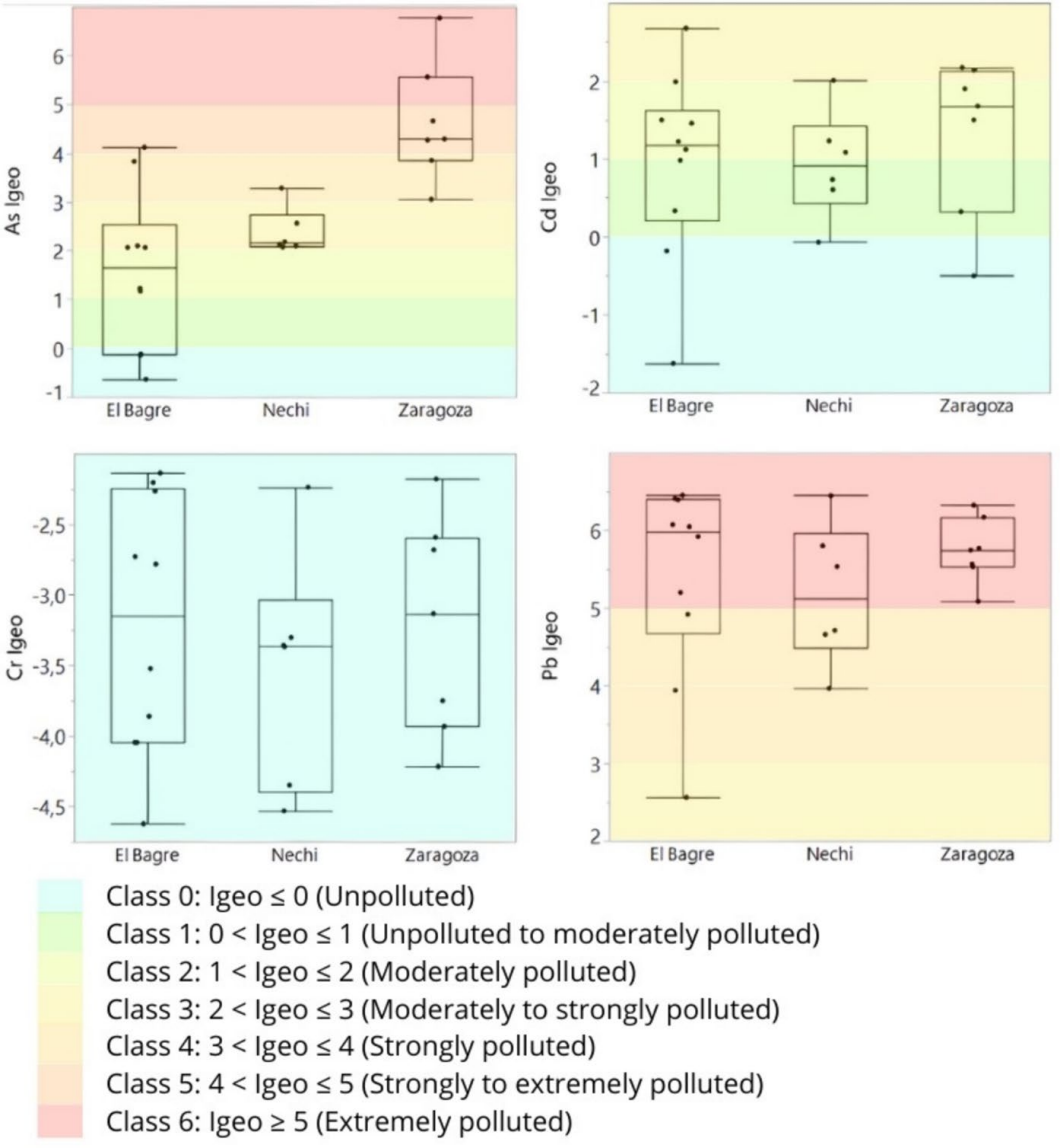
Geoaccumulation Index (Igeo) in the municipalities of El Bagre, Nechi, and Zaragoza, Antioquia, Colombia

The results of the Igeo are shown in Fig. 4. In the municipality of Zaragoza, a significant enrichment of As was observed, with Igeo values greater than 5. Pb was categorized as extremely contaminated, with values above 5 in most of the samples from the three municipalities. In the case of Cd, the Igeo values ranged from low to moderately contaminated, with values between 0 and 2 in the majority of the samples. For Cr, no contamination process was identified, since all the samples from the three municipalities showed values below 0. The extreme contamination levels indicated for As and Pb are possibly associated with geogenic sources present in the area (Salazar et al., 2024). Furthermore, the observed Igeo values may also be associated with the weathering, leaching, and subsequent redistribution of PTE-enriched materials derived from the surrounding environment. The background concentration for As and Pb in the study region average ~ 12.2 and ~ 22.8 mg kg^−1^, respectively (López et al., 2025). It is important to note that background concentrations in mineralized areas may be naturally higher. Therefore, the use of the Colombian background values, as applied in this study, may lead to an overestimation of the Igeo index. Consequently, the results and interpretations derived from the Igeo index should be considered in light of this limitation.

### Potential ecological risk index

**Fig. 5:**
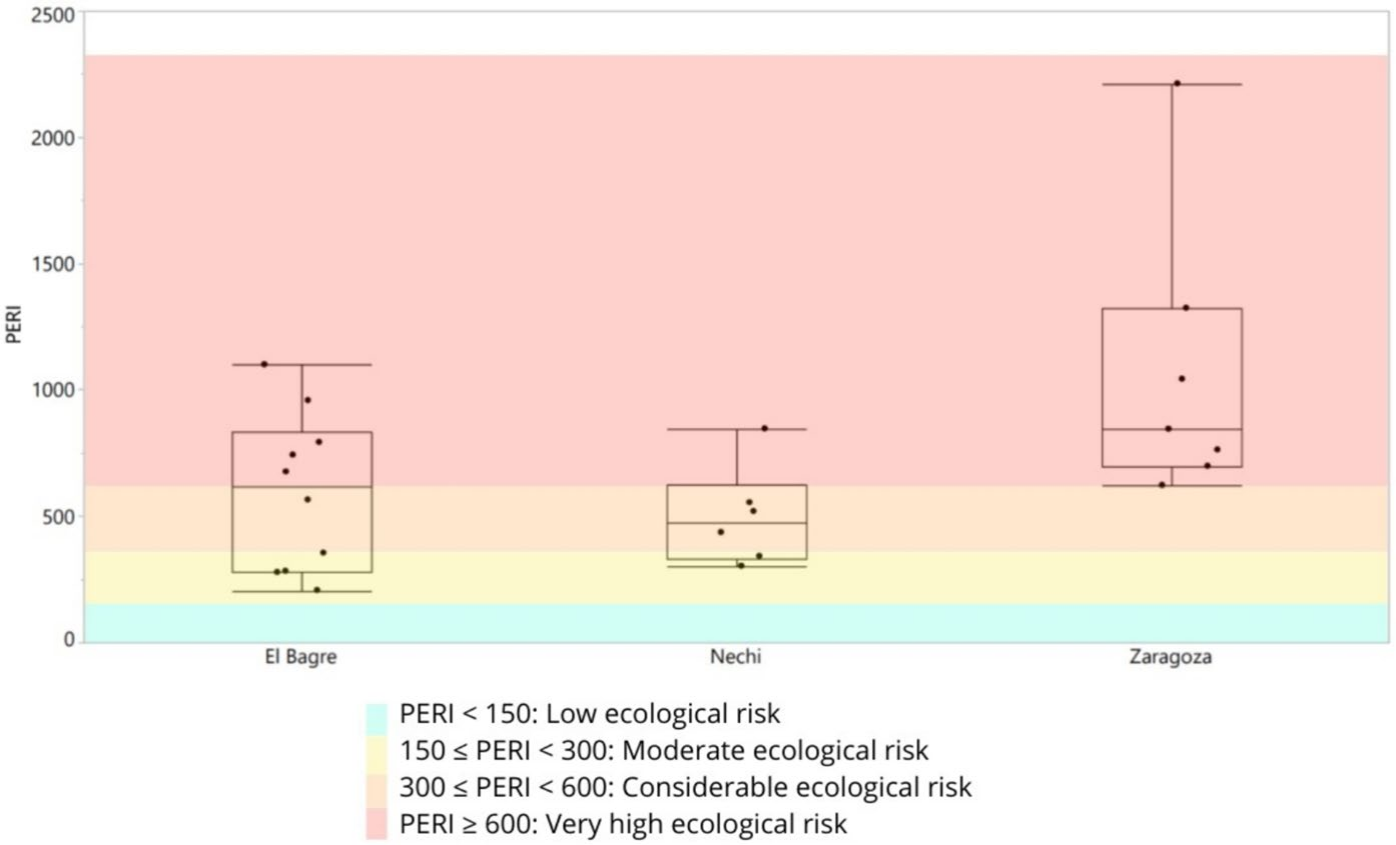
Potential ecological risk index (PERI) in the municipalities of El Bagre, Nechi, and Zaragoza, Antioquia, Colombia

The results of the PERI calculation are shown in Fig. 5. Zaragoza had the highest PERI values, followed by El Bagre and Nechí. The mean PERI values for Zaragoza, El Bagre, and Nechí were ~ 800, ~ 600, and ~ 450, respectively. PERI values ranged from ~ 800 to ~ 2,200 in Zaragoza, ~ 200 to ~ 1,100 in El Bagre, and ~ 250 to ~ 800 in Nechí. Based on the proposed PERI classification (Hakanson, 1980), in Zaragoza, 15.8% of the values were classified as considerable risk and 84.6% as high risk. In El Bagre, 10% of the values were classified as considerable risk and 90% as high risk. In Nechí, 15% and 85% of the values were classified as considerable and high risk, respectively. None of the values were categorized as low or moderate risk. These results indicate that, considering the toxicological response values (Hakanson, 1980) for each of the PTEs used in the PERI estimation, the concentrations of PTEs determined in Zaragoza, El Bagre, and Nechí represent a significant potential risk ranging from considerable to high for the ecosystems.

### Categorization and prioritization of environmental liabilities

**Fig. 6:**
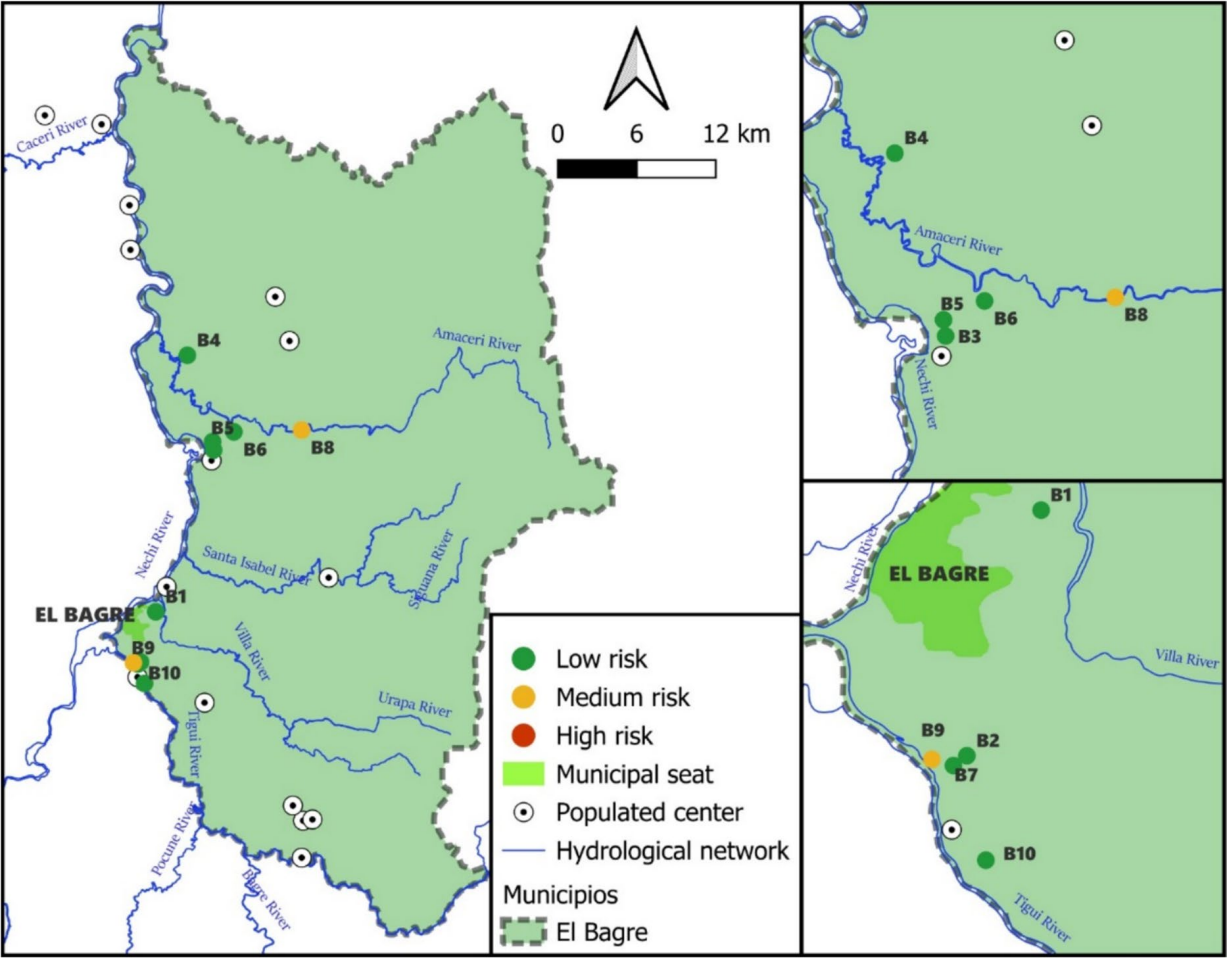
Point map showing the spatial distribution of mine tailings, classified according to the risk assessment (RI) methodology, in the municipalities of El Bagre, Antioquia, Colombia

**Fig. 7:**
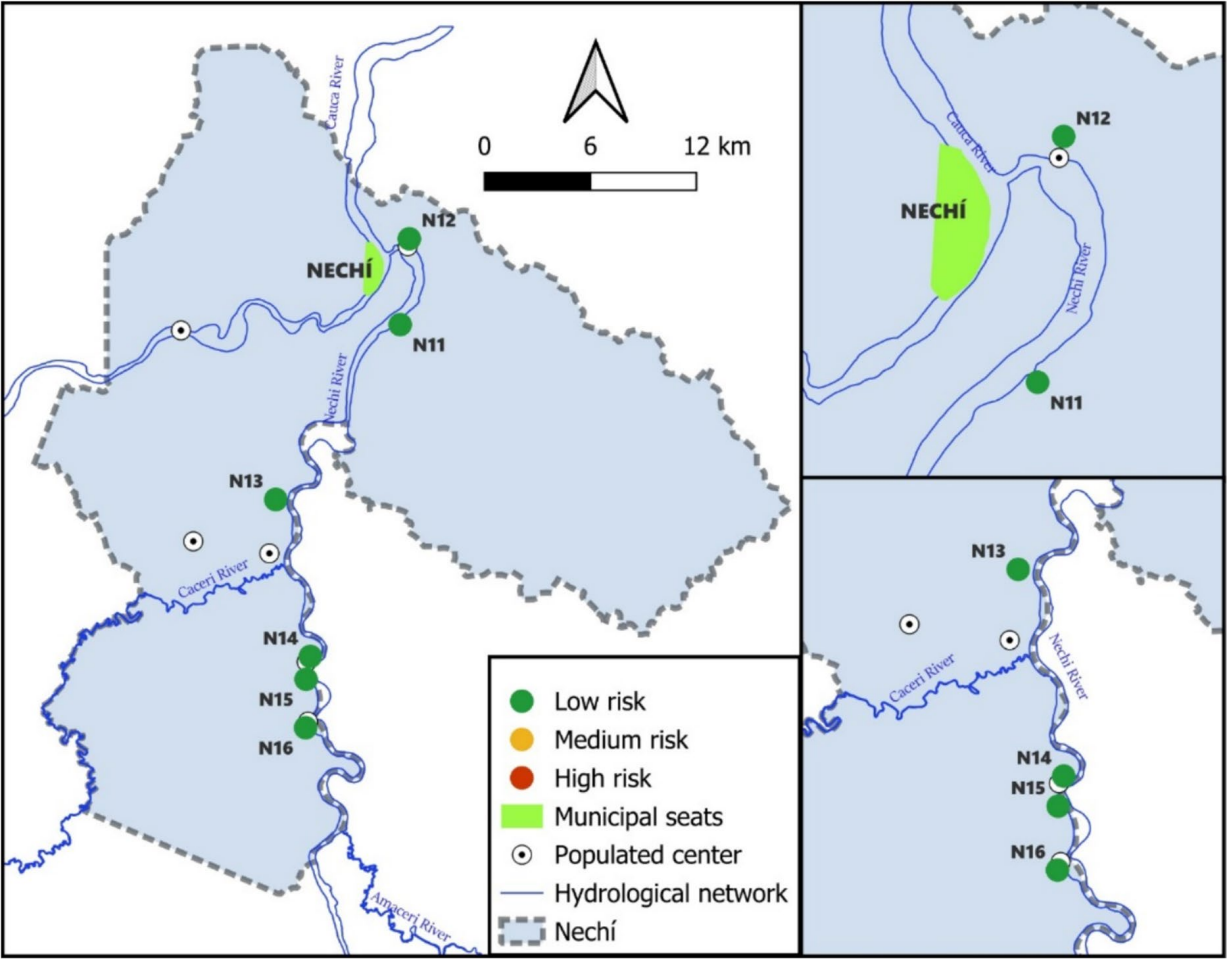
Point map showing the spatial distribution of mine tailings, classified according to the risk assessment (RI) methodology, in the municipalities of Nechi, Antioquia, Colombia

**Fig. 8:**
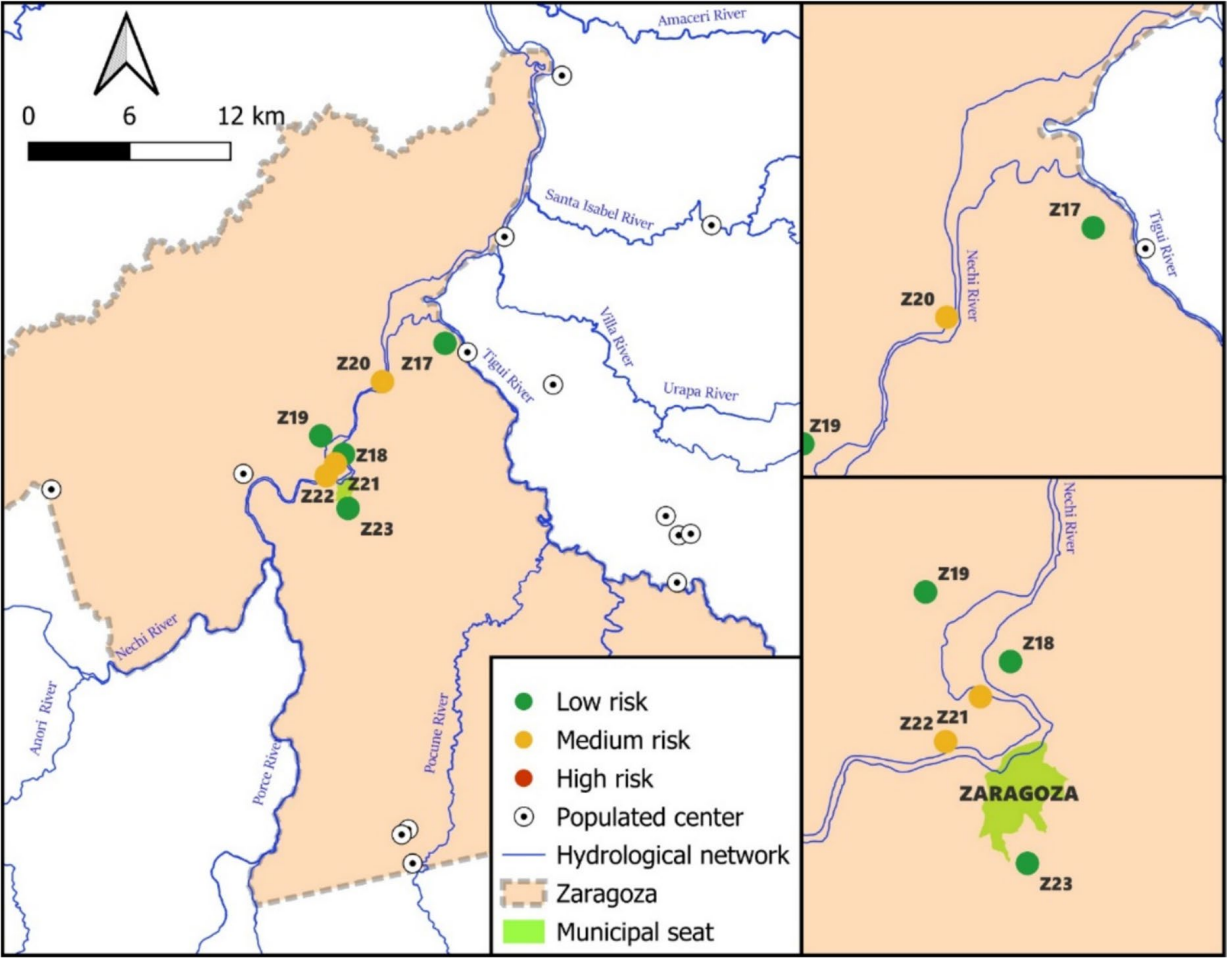
Point map showing the spatial distribution of mine tailings, classified according to the risk assessment (RI) methodology, in the municipalities of Zaragoza, Antioquia, Colombia

For the categorization and prioritization of environmental liabilities, the values proposed by the Spanish Geological Survey were used, as applied in similar studies such as Salgado-Almeida et al. (2024). The RI classification was as follows: low risk (0 ≤ RI ≤ 5), moderate risk (6 ≤ RI ≤ 15), and high risk (16 ≤ RI ≤ 25). In the municipality of El Bagre, 20% of the samples were classified as having a medium RI and 80% as having a low RI (Fig. 6). In Nechí, 100% of the samples were classified as having a low RI (Fig. 7). In Zaragoza, 43% of the samples were classified as having a medium RI and 57% as having a low RI (Fig. 8). None of the three municipalities recorded samples with a high RI classification. The results of the categorization of environmental liabilities according to the RI generally indicate a moderate to low risk, which contrasts with the analysis and interpretation of PTEs concentration values and the Igeo and PERI indices, which revealed significant contamination processes. This makes it clear that factors such as the distance of tailings deposits from water bodies and populated areas must be considered when assessing the risk associated with environmental liabilities. The evaluation should not rely solely on PTEs concentrations as the only categorization criterion, nor on the calculation of an index that considers only the relationship between PTEs concentrations in the samples and the local background values. These results are consistent with the reports by (López et al., 2024b), which suggest that, for prioritizing the treatment of soils impacted by gold mining, it is necessary to develop a multi-criteria framework that incorporates factors beyond PTEs concentration values.

The RI results therefore make it possible to prioritize two environmental liabilities in the municipality of El Bagre and three in the municipality of Zaragoza. These should be addressed for environmental management by the relevant stakeholders. In these areas, the population potentially exposed to risk ranges from 28,000 inhabitants in Zaragoza to 55,000 inhabitants in El Bagre, respectively. In this subregion, many people use water bodies for recreational purposes and fishing activities, which increases the likelihood of human exposure to PTEs through pathways such as accidental ingestion while drinking from these sources during recreational use, as well as through dermal contact. For instance, López et al. (2025) found that in municipalities near the Bajo Cauca region of Antioquia, the human health risk associated with exposure to PTEs through ingestion and dermal contact in gold mining–impacted areas were significant. For the same region, studies have documented phytotoxic effects in plant models exposed to soil samples collected from contaminated areas affected by illegal mining activities (López et al., 2024a, 2024b). Furthermore, the consumption of fish caught in these waters may lead to biomagnification processes of PTEs. Studies conducted in La Mojana, a subregion adjacent to the Bajo Cauca region of Antioquia, have reported the presence of PTEs in hair and blood samples from children (Palomares-Bolaños et al., 2025). Other studies conducted north of the Bajo Cauca region of Antioquia have identified a relationship between gold mining activities, Hg contamination, bioaccumulation in fish consumed as food, and human exposure within local populations (Olivero-Verbel et al., 2011).

The implementation of management measures for these tailings’ deposits will therefore be necessary in order to minimize risks to nearby populations and to prevent soil and water contamination in the surrounding areas. These alternatives may include the valorization of mining tailings as construction materials. Tailings have been reused to produce bricks, ceramic materials, and cement (Hajdu-Rahkama & Kinnunen, 2025). Ecosystem restoration through the control of PTEs contamination and revegetation also represents a viable option (López et al., 2024b; Vargas et al., 2024; Wu et al., 2024). In addition, continuous environmental monitoring in these prioritized areas should be a critical component, requiring the use of both physicochemical and biological environmental indicators (López et al., 2024a, 2024b).

## Public policy

In Colombia, since 2015, the need for a strategy to identify, assess, and manage environmental liabilities has been recognized. Subsequently, in 2016, a proposal was developed outlining the technical instruments necessary for the management of environmental liabilities across the national territory. This was followed in 2017 by the introduction of an economic and financial strategy for managing environmental liabilities. In 2018, the first initiative emerged to develop a methodological framework to allow for the prioritization of environmental liability management, which in 2020 was complemented by the conceptualization of the Environmental Liabilities Information System. The results of this study provide scientific evidence that will contribute to the development of a methodology for the categorization and prioritization of environmental liabilities associated with gold mining. Overall, the findings of this study provide valuable scientific evidence to support the formulation of public policies and decision-making processes.

## Conclusions

The implementation of multi-criteria frameworks, ecosystem restoration, tailings reclamation, and continuous environmental monitoring is crucial to mitigate risks to human health and ecosystems while strengthening decision-making based on robust scientific and technical evidence. This study presents the results of applying a methodology for the categorization and prioritization of environmental liabilities associated with gold mining. A first conclusion is related to As as a relevant PTEs, especially in the municipality of Zaragoza. Concentration of this element can be used as an indicator of contamination in the studied mining tailings. Another conclusion is derived from the application of three indices: the Risk Index, the Igeo index, and the PERI index. On one hand, the evaluation of environmental liabilities in the municipalities of El Bagre, Nechí, and Zaragoza shows that most samples are classified as low to medium risk according to the Risk Index. On the other hand, the use of indices such as Igeo and PERI shows results that classify the contamination process by As, Pb, Cd, and Cr as significant pollution. It can therefore be concluded that there is a discrepancy between the classification obtained from the Risk Index and those derived from the Igeo and PERI indices. This difference is mainly explained by the fact that the former includes territorial variables such as distance to population centers and key environmental features of the territory, such as rivers. This implies that the Risk Index does not only consider the concentration of PTEs, but also relates it to the probability of exposure to these elements. In light of these results, the Risk Index may serve as a useful reference framework for decision-making and management prioritization. Further studies will be necessary to strengthen the information regarding carcinogenic and non-carcinogenic risks associated with PTEs exposure in the area, using deterministic and probabilistic methods, as well as the inclusion of bioassays to obtain results on the responses of biological models to these contamination levels.

## Supplementary Information

Below is the link to the electronic supplementary material.

Supplementary file1 (DOCX 1468 KB)

## Data Availability

Data will be made available on request.
