# Supplementary material for: Assessment of potentially toxic elements in mine tailings and their categorization and prioritization as environmental liabilities in gold mining areas of Antioquia, Colombia
Source: Environ Geochem Health. 2026 Jul 10;48(10):444. doi: 10.1007/s10653-026-03322-5 (PMC13354650; doi:10.1007/s10653-026-03322-5)
Supplement: Supplementary file 1 — Supplementary file1 (DOCX 1468 KB) [file 10653_2026_3322_MOESM1_ESM.docx]

**Supplementary information for:**

**Assessment of Potentially Toxic Elements in Mine Tailings and Their Categorization and Prioritization as Environmental Liabilities in Gold Mining Areas of Antioquia, Colombia**

Sebastian Collazo^1a^, Esteban Vélez^1a^, Juan D. Correa^1^, Juan F. Marín^1^, Juan F. Saldarriaga^2^, Julián E. López^1*^

^1^ Faculty of Architecture and Engineering, Study Group SICA-GeoHealth, Semillero de investigación SICA, Institución Universitaria Colegio Mayor de Antioquia, Carrera 78 # 65 – 46, 050034, Medellín, Colombia

^2^ Department of Civil and Environmental Engineering, Universidad de los Andes, Carrera 1Este #19A-40, 111711, Bogotá, Colombia

^a^ Joint first authors, all contributed equally

***Corresponding author**

Julián E. López

julian.lopez@colmayor.edu.co


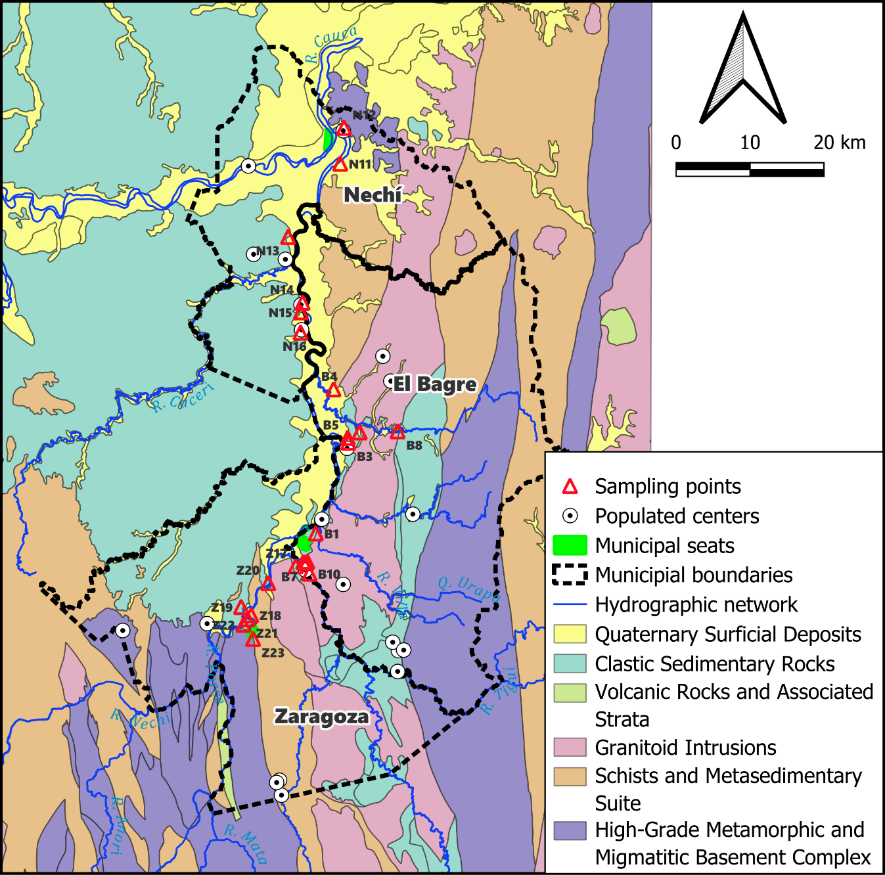


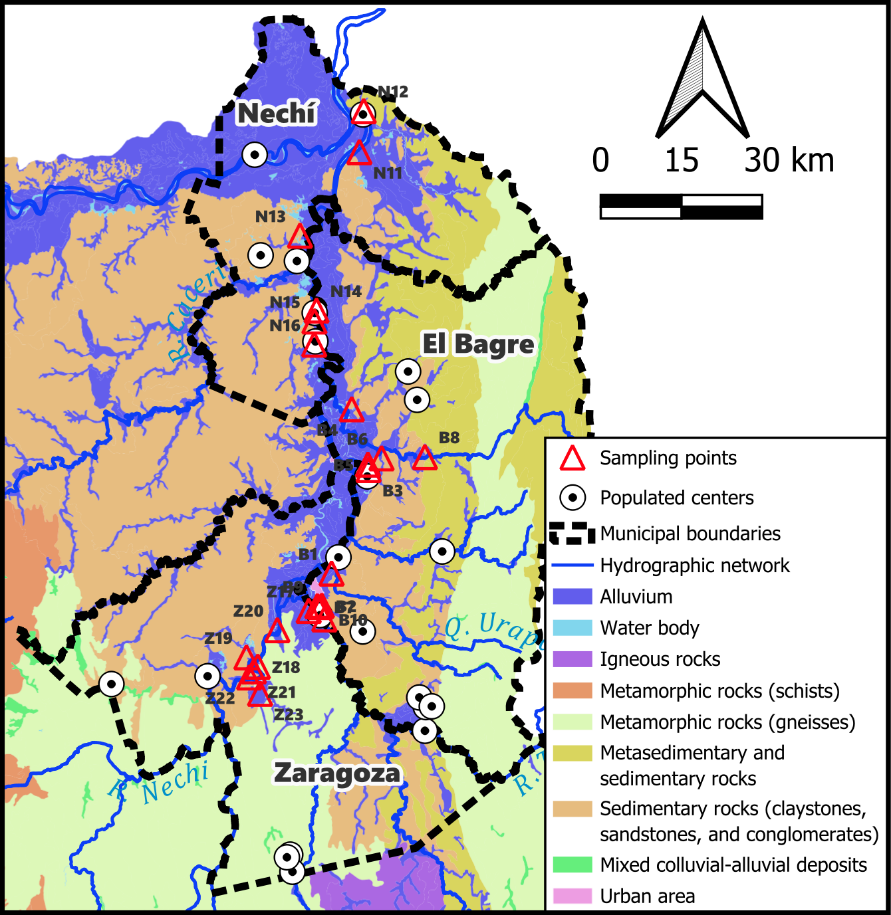


**Fig** S1. Geological / Lithological map of the study area.


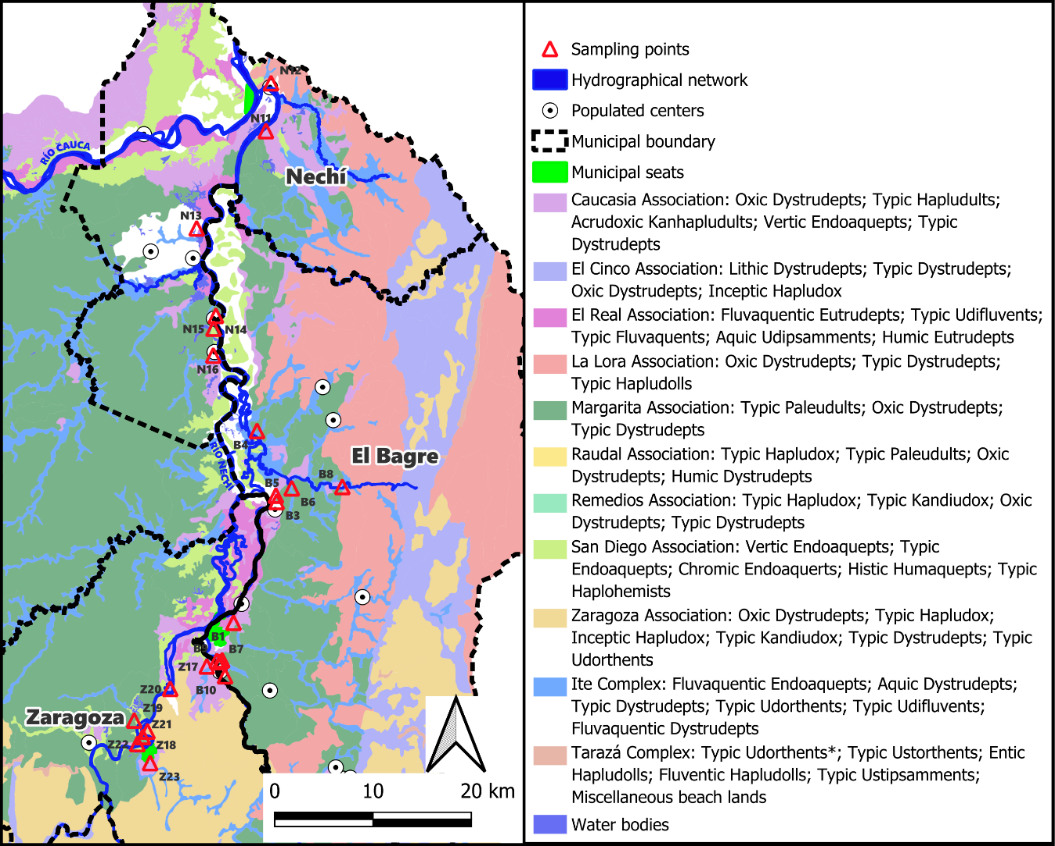


**Fig** S2. Soil map of the study area.

Table S1. Concentration of PTEs in gold mining tailings.

| Location | Ore | As  (mg kg-1) | Cd  (mg kg-1) | Pb  (mg kg-1) | Cr  (mg kg-1) | References |
| --- | --- | --- | --- | --- | --- | --- |
| Tanzania* | Au | 6.17-1399.2 (702) | 4.45-11.83 (8.14) | 64.56-101.4 (82.98) | 155-426.3 (290) | (Kaaya et al., 2025) |
| Ecuador* | Au | 429-4466  (2049) | 0.2-5.18  (2.1) | 2.73-410  (133) | 143-268  (218) | (Garcés et al., 2025) |
| Peru* | Au | 109-6653  (2354) | 4.2-930  (293) | 51.1-11250  (4559) | - | (Hammer et al., 2024) |
| Colombia* | Au | 27-3555 (191) | 3.39-65.2 (65.6) | 37-9726 (4881) | 13-281  (147) | (Salazar et al., 2024) |
| Ecuador* | Au | 23.6-5772  (1964) | 0.2-96.2  (96.2) | 37.4-6196  (1201) | 25.1-88.3  (49.9) | (Salgado-Almeida et al., 2024) |
| Ghana*** | Au | 19 | 3.1 | 14 | - | (Akoto & Anning, 2021) |
| Namibia* | Au, Cu | - | - | 19-183 (101) | 10-95 (52.5) | (Uugwanga & Kgabi, 2020) |
| Brazil*** | Au | 413  ± 4 | 143  ± 4 | 23337  ± 4256 | 413  ± 4 | (Barcelos et al., 2020) |
| Nigeria | Au | 4.79 | 0.68 | 2802 | 96.9 | (Adewumi & Laniyan, 2020) |
| Canada* | Au | 57.5-766.4 (350.4) | - | 7.05-31.31 (17,2) | 1.52-4.36 (2.7) | (Gagnon et al., 2020) |
| Chile* | Au, Cu | 10.1-119 (64.5) | - | 15-272 (143) |  | (Medina Tripodi et al., 2019) |
| Egypt* | Au | 2643-3183 (2936.3) | - | 11.8-16 (9) | 224-244 (237) | (Redwan & Bamousa, 2019) |
| Finlanda*** | Au | 1520 | <0.3 | 4.3 | 74 | (Kiventerä et al., 2018) |
| China* | Au | 0.15-2.28 (0.89) | 2.39-16  (7) | 599-2105 (599) | - | (Xiao et al., 2017) |
| Cuba* | Au | 1085 - 8650 (5410) | - | - | - | (Toujaguez et al., 2013) |
| Korea* | Au, Ag | 143.8-3584 (51.4) | 2.2-20 (9.4) | 125-50.8 (20.3) | - | (Lim et al., 2008)) |
| Kongo* | Au | 1787-4629 (3208) | - | - | - | (Nyarko, 2001) |

PTEs: potential toxic elements. *Values are represented as Minimum-Maximum (Mean). ^**^ Concentration of PTEs ± Standard deviation. *** Only a single value was reported.

**References**

Adewumi, A. J., & Laniyan, T. A. (2020). Contamination, sources and risk assessments of metals in media from Anka artisanal gold mining area, Northwest Nigeria. *Science of The Total Environment*, *718*, 137235. https://doi.org/10.1016/j.scitotenv.2020.137235

Akoto, R., & Anning, A. K. (2021). Heavy metal enrichment and potential ecological risks from different solid mine wastes at a mine site in Ghana. *Environmental Advances*, *3*, 100028. https://doi.org/10.1016/j.envadv.2020.100028

Barcelos, D. A., Pontes, F. V. M., da Silva, F. A. N. G., Castro, D. C., dos Anjos, N. O. A., & Castilhos, Z. C. (2020). Gold mining tailing: Environmental availability of metals and human health risk assessment. *Journal of Hazardous Materials*, *397*, 122721. https://doi.org/10.1016/j.jhazmat.2020.122721

Gagnon, V., Rodrigue-Morin, M., Migneault, M., Tardif, A., Garneau, L., Lalonde, S., Shipley, B., Greer, C. W., Bellenger, J.-P., & Roy, S. (2020). Survival, growth and element translocation by 4 plant species growing on acidogenic gold mine tailings in Québec. *Ecological Engineering*, *151*, 105855. https://doi.org/10.1016/j.ecoleng.2020.105855

Garcés, D., Jiménez-Oyola, S., Sánchez-Palencia, Y., Guzmán-Martínez, F., Villavicencio-Espinoza, R., Jaramillo-Zambrano, S., Rosado, V., Salgado-Almeida, B., & Marcillo-Guillén, J. (2025). Assessment of Tailings Contamination Potential in One of the Most Important Gold Mining Districts of Ecuador. *Minerals*, *15*(8), 767. https://doi.org/10.3390/min15080767

Hammer, V., Vanneste, J., Alejo-Zapata, F. D., Zea, J., Bolaños-Sosa, H. G., Zevallos Rojas, C. A., Figueroa, L. A., Malone, A., Bellona, C., & Vuono, D. C. (2024). Characterization of medium and small-scale gold processing operations, wastewaters, and tailings in the Arequipa region of Peru. *Science of The Total Environment*, *945*, 174034. https://doi.org/10.1016/j.scitotenv.2024.174034

Kaaya, N. I., Vegi, M. R., & Macheyeki, A. S. (2025). Health risks of geogenic contaminants in gold mining areas in Geita, Tanzania. *Journal of Trace Elements and Minerals*, *12*, 100222. https://doi.org/10.1016/j.jtemin.2025.100222

Kiventerä, J., Sreenivasan, H., Cheeseman, C., Kinnunen, P., & Illikainen, M. (2018). Immobilization of sulfates and heavy metals in gold mine tailings by sodium silicate and hydrated lime. *Journal of Environmental Chemical Engineering*, *6*(5), 6530–6536. https://doi.org/10.1016/j.jece.2018.10.012

Lim, H.-S., Lee, J.-S., Chon, H.-T., & Sager, M. (2008). Heavy metal contamination and health risk assessment in the vicinity of the abandoned Songcheon Au–Ag mine in Korea. *Journal of Geochemical Exploration*, *96*(2–3), 223–230. https://doi.org/10.1016/j.gexplo.2007.04.008

Medina Tripodi, E. E., Gamboa Rueda, J. A., Aguirre Céspedes, C., Delgado Vega, J., & Collao Gómez, C. (2019). Characterization and geostatistical modelling of contaminants and added value metals from an abandoned Cu–Au tailing dam in Taltal (Chile). *Journal of South American Earth Sciences*, *93*, 183–202. https://doi.org/10.1016/j.jsames.2019.05.001

Nyarko, B. J. B. (2001). Determination of arsenic in some water bodies, untreated ore and tailing samples at Konongo in the Ashanti region of Ghana and its surrounding towns and villages by instrumental neutron activation analysis. *Journal of Radioanalytical and Nuclear Chemistry*, *249*(3), 581–585. https://doi.org/10.1023/A:1013246231569

Redwan, M., & Bamousa, A. O. (2019). Characterization and environmental impact assessment of gold mine tailings in arid regions: A case study of Barramiya gold mine area, Eastern Desert, Egypt. *Journal of African Earth Sciences*, *160*, 103644. https://doi.org/10.1016/j.jafrearsci.2019.103644

Salgado-Almeida, B., Briones-Escalante, A., Falquez-Torres, D., Filián-Haz, K., Guzmán-Martínez, F., Escobar-Segovia, K., Peña-Carpio, E., & Jiménez-Oyola, S. (2024). Assessment of Environmental Pollution and Risks Associated with Tailing Dams in a Historical Gold Mining Area of Ecuador. *Resources*, *13*(8), 105. https://doi.org/10.3390/resources13080105

Salazar, J. P., Saldarriaga, J. F., Zapata, D., & López, J. E. (2024). Determination of Bioavailability, Potential Ecological and Human Health Risks, and Biomonitoring of Potential Toxic Elements in Gold Mine Tailings from Four Areas of Antioquia, Colombia. Water, Air, & Soil Pollution, 235(2), 122. https://doi.org/10.1007/s11270-024-06893-0

Toujaguez, R., Ono, F. B., Martins, V., Cabrera, P. P., Blanco, A. V., Bundschuh, J., & Guilherme, L. R. G. (2013). Arsenic bioaccessibility in gold mine tailings of Delita, Cuba. *Journal of Hazardous Materials*, *262*, 1004–1013. https://doi.org/10.1016/j.jhazmat.2013.01.045

Uugwanga, M. N., & Kgabi, N. A. (2020). Assessment of metals pollution in sediments and tailings of Klein Aub and Oamites mine sites, Namibia. *Environmental Advances*, *2*, 100006. https://doi.org/10.1016/j.envadv.2020.100006

Xiao, R., Wang, S., Li, R., Wang, J. J., & Zhang, Z. (2017). Soil heavy metal contamination and health risks associated with artisanal gold mining in Tongguan, Shaanxi, China. *Ecotoxicology and Environmental Safety*, *141*, 17–24. https://doi.org/10.1016/j.ecoenv.2017.03.002
